# Concurrent visual sequence learning

**DOI:** 10.1007/s00426-023-01810-2

**Published:** 2023-03-22

**Authors:** Sarah Wilts, Hilde Haider

**Affiliations:** Department of Psychology, University of Cologne, Richard-Strauss-Str. 2, 50931 Cologne, Germany

## Abstract

Many researchers in the field of implicit statistical learning agree that there does not exist one general implicit learning mechanism, but rather, that implicit learning takes place in highly specialized encapsulated modules. However, the exact representational content of these modules is still under debate. While there is ample evidence for a distinction between modalities (e.g., visual, auditory perception), the representational content of the modules might even be distinguished by features within the same modalities (e.g., location, color, and shape within the visual modality). In implicit sequence learning, there is evidence for the latter hypothesis, as a stimulus-color sequence can be learned concurrently with a stimulus-location sequence. Our aim was to test whether this also holds true for non-spatial features within the visual modality. This has been shown in artificial grammar learning, but not yet in implicit sequence learning. Hence, in Experiment 1, we replicated an artificial grammar learning experiment of Conway and Christiansen (2006) in which participants were supposed to learn color and shape grammars concurrently. In Experiment 2, we investigated concurrent learning of sequences with an implicit sequence learning paradigm: the serial reaction time task. Here, we found evidence for concurrent learning of two sequences, a color and shape sequence. Overall, the findings converge to the assumption that implicit learning might be based on features.

**Supplementary Information:**

The online version contains supplementary material available at 10.1007/s00426-023-01810-2.

## Introduction

Learning is a fundamental ability enabling humans to adapt to almost every new situation in everyday life. Often, such skills are acquired by simply observing or repeating actions even without being aware of any learning processes or of the underlying rules and structures having been learnt (Reber, 1967). For instance, preschoolers learn their first language incidentally simply by listening, but cannot articulate the underlying rules (Perruchet et al., 2002). In a similar vein, students learn to type fluently and even blindly on a computer keyboard without knowing explicitly which letter occurs at which location (Snyder et al., 2013). More generally, humans acquire regularities inherent in the environment without any effort or intention to learn. Further examples are driving, making dinner, or practicing musical instruments (Geiger et al., 2018). This kind of learning is usually termed implicit learning or implicit statistical learning (we use these terms synonymously; Conway & Christiansen, 2006; Perruchet & Pacton, 2006).

Despite the frequent use of the term implicit learning in slightly different and only loosely connected fields, it is by no means clear whether it refers to one and the same learning mechanism or to different learning mechanisms (see Christiansen, 2019, for an alignment of different methodologies in implicit statistical learning). In what follows, we focus on the question: What are the (minimal) building blocks of implicit learning? The current study aims at investigating this question within two different paradigms, Artificial Grammar Learning (AGL; Reber, 1967) and the Serial Reaction Time Task (SRTT; Nissen & Bullemer, 1987), to test for commonalities between these two paradigms.

We start with a brief introduction of the typical AGL and SRTT paradigms. We then discuss theoretical assumptions about the building blocks of implicit learning and report afterwards empirical findings that already provide some hints about the characteristics of the representations underlying implicit learning.

### Research paradigms in implicit learning

In the standard AGL experiment (Reber, 1967), the participants are asked to observe short strings of stimuli that are, unbeknownst to them, derived from a complex set of rules, the grammar. Afterwards a test phase follows. The participants receive new strings that either follow or do not follow the grammar and they have to judge whether a given string is grammatical or ungrammatical. The usual finding is that the participants judge the grammaticality of the strings better than chance-level, but concurrently are not able to explicate the underlying rules. Albeit debatable, Reber (1967) concluded from these findings that learning of the grammar is implicit. For instance, Perruchet and Pacton (1990) provided evidence that implicit grammar learning might be based on explicit knowledge about grammar fragments (e.g., single bigrams).

In contrast to the AGL, in the standard SRTT originated by Nissen and Bullemer (1987), the participants see several locations marked on the screen. These screen-locations are spatially mapped to response keys. In each trial, an asterisk (target) appears at one of the marked screen locations and the participants are asked to press the assigned response key as quickly and accurately as possible. Unbeknownst to the participants, the locations of the asterisk follow a regular sequence. This sequence is replaced by a random sequence after a few practice blocks and is re-introduced thereafter. This leads to an increase in response times and/or error rates which disappears as soon as the regular sequence reoccurs. Analogous to the AGL, most participants are unable to explicate their acquired sequence knowledge. Meanwhile, the standard SRTT has been modified to assess not only perceptual-motor learning, but also, for instance, pure perceptual learning or the concurrent learning of uncorrelated sequences (e.g., perceptual sequences: stimulus-color sequence, stimulus-location sequence; or a motor sequence: response-location sequence; Eberhardt et al., 2017, Goschke & Bolte, 2012; Haider et al., 2012; Haider et al., 2014; Howard et al., 1992; Mayr, 1996; Remillard, 2009, 2011).

### Assumptions concerning the building blocks of implicit learning

Even though such additional variations of the SRTT might have attenuated the boundaries between the research topics investigated within the fields of the AGL and the SRTT experiments, the two fields are only loosely connected (Frost et al., 2015). This is somewhat surprising since the research questions in both fields are quite similar. In both fields, a central question concerns the building blocks of implicit learning (Conway, 2020; Conway & Christiansen, 2006; Eberhardt et al., 2017; Goschke & Bolte, 2012; Haider et al., 2018; Mayr, 1996). Yet, there is no agreement on what exactly the processed contents might be that become associated within implicit learning tasks. For instance, implicit learning might refer to contents represented in different modalities like vision, or audition (Abrahamse et al., 2010; Frost et al., 2015). Yet, alternatively, some authors now propose to look at a more fine-grained level within modalities, namely features like color, shape, or location for the visual modality which form the smallest entities that can become associated in an implicit learning task (e.g., Conway & Christiansen, 2006; Eberhardt et al., 2017; Haider et al., 2018). It is important to note that the conceptualization of features, as we use it here, stems from the Theory of Event Coding (TEC; Hommel, 2009; Hommel et al., 2001), a theory of perception and action planning. In short, according to the TEC, it is assumed that both perceptions and actions are represented in terms of distal events in the form of consciously available feature codes. These sensorimotor feature codes, for instance the feature code “left” as part of the feature “location” are made up of various proximal sensory and motor representations. These representations are distributed all over the cortex, such as color, shape, or location are represented in distinct parts of the visual cortex (Hommel, 2009). To plan or conduct an action means that such feature codes are bound together in so-called event files (Hommel, 2004). We, therefore, assume that implicit learning might be based on associations between such feature codes belonging to one feature. This would imply that two regularities can be learned concurrently as long as they belong to different features.

Such an account concerning the representations underlying implicit learning might help to better understand implicit learning. When looking at the theoretical accounts, it becomes obvious that in both fields the proposals only loosely define the characteristics of the representations underlying implicit learning. For instance, in SRTT learning, Keele and colleagues (2003) proposed the Dual-System Model.^1^ In this model, the crucial assumption is that implicit learning in the unidimensional system takes place in multiple encapsulated modules which are each specialized to process information along a particular dimension. Due to this specificity, the modules can process information in parallel as long as they belong to different dimensions.

Thus, according to the Dual-System Model, it is the particular dimension processed in the unidimensional system that constitutes the building blocks of implicitly learned sequences. As already noticed by the authors themselves, the problem is that the term dimension is underspecified. It may refer to modalities, like vision, audition and so on. Alternatively, it might refer to features within modalities, like color, shape, and location.

In the field of AGL, Frost et al., (2015, 2019) proposed in their model of Perceptual Statistical Learning to interpret implicit learning as an interaction between domain-specific and domain-general learning processes. Domain-specific learning takes place in different cortical areas (e.g., learning of a visual regularity in the visual cortex). Hence, learning of specific regularities is constrained by properties of the respective cortices and therefore, modality-specific. However, the encoding of specific stimuli within a modality can also take place in different brain regions leading to stimulus-specific representations (e.g., learning of a regularity of colors in a specific part of the visual cortex). These representations are fed into a multi-modal region for further domain-general computations in the medial temporal lobe memory system (Frost et al., 2015).

According to Frost et al., (2015), the capacity of the domain-specific learning processes is limited. Factors, such as the complexity and similarity of the to-be-associated material, influence whether learning is modality- or stimulus-specific. These factors remain underspecified by the authors. We propose that two regularities instantiated by two distinct features (e.g., color, shape, etc.) might be processed concurrently and independently from each other.

Recently, Conway (2020) combined the implicit and explicit learning modes from Keele et al., (2003) with modality-specific and domain-general learning mechanisms similar to Frost et al., (2015) and also arrived at the question how learning might be affected by the input domain.

In summary, the exact understanding of the terms dimension or domain remains unclear in the above-described models and hence, the question about the content of the representations underlying implicit learning is still unanswered (Frost et al., 2015; Keele et al., 2003). We propose to equate the terms dimension and domain with feature, such as colors, shapes, and location in the visual domain.

### Empirical evidence

A usual way to investigate the (minimal) building blocks of implicit learning is to train participants in an implicit learning task with two uncorrelated statistical regularities (Abrahamse et al., 2010; Conway & Christiansen, 2006; Goschke & Bolte, 2012). If the participants can successfully acquire these two regularities concurrently, the participants must have been able to keep the representations of the statistical regularities separate. A few studies investigated already this parallel learning of multiple regularities within AGL and SRTT designs (i.e., Conway & Christiansen, 2006; Deroost & Soetens, 2006; Li et al., 2018; Mayr, 1996; Witt & Willingham, 2006). The research can be summarized with regard to learning of multiple regularities that were instantiated in either different or the same modality, and in either different or the same feature.

### Learning of regularities based on two different features in separate modalities

As one of the first who investigated learning in two different modalities in the SRTT, Mayr (1996) trained participants to discriminate object-identities by pressing the assigned keys. The objects appeared in one of the four corners of a square on the screen. Importantly, both the object-identities and the screen-locations followed uncorrelated sequences, respectively. The findings confirmed that participants could learn the response (object-identities) and the perceptual sequence (screen-locations) simultaneously. Deroost and Soetens (2006) replicated this SRTT experiment, but additionally showed that small changes in the experimental design hampered already the learning of the perceptual sequence. Thus, it seems that simultaneous learning of a perceptual and a response sequence is possible, even though perceptual learning seems to be rather fragile (Rüsseler & Rösler, 2000).

In a modified AGL experiment of Conway and Christiansen (2006), the participants received randomly intermixed visual color strings and auditory tone strings that were both derived from two different grammars. The authors observed simultaneous learning of both grammars and concluded that concurrent learning of two grammars instantiated by two different modalities (visual versus auditory) is possible.

### Learning of regularities based on two different features within the same modality

Goschke and Bolte (2012) succeeded in showing that participants could learn two uncorrelated perceptual sequences (one consisting of stimulus-locations, the other of visual target-letters) concurrently with a third uncorrelated response-location sequence in the SRTT. First, their results support the findings of Mayr (1996) that a perceptual and a response sequence can be learned concurrently. Furthermore, they showed that two sequences within the same modality (visual) instantiated by the features location and shape can also be learned concurrently.

Similarly, in the AGL, Conway and Christiansen (2006) showed that participants learned two grammars presented by two perceptual features (color and shape) concurrently. This result has also been replicated by Johansson (2009). Furthermore, Turk-Browne et al. (2008) showed that visual strings of colors and shapes can be learned concurrently in the AGL, even when they were displayed in the same object, as long as the regularities did not co-vary. Walk and Conway (2016) also provided evidence for concurrent learning of color and shape strings derived from two different sets of grammars.

### When the concurrent learning of two regularities fails

In the already mentioned modified AGL design of Conway and Christiansen (2006), the authors also tested for learning of two grammars instantiated by the same perceptual feature (two sets of shape strings). In this condition, the participants were unable to learn the grammatical rules of the two shape sets. Thus, if the strings derived from different grammars are based on the same feature, simultaneous learning of grammatical rules is impossible. In the SRTT, Eberhardt et al., (2017) went one step further by asking for the constraints of concurrently learning two uncorrelated sequences in parallel. In their study, the participants received either a visual-color or a response-location sequence together with a sequence of screen-locations. The results revealed that while the participants learned the visual-color sequence simultaneously with the screen-location sequence (both sequences were perceptual sequences), they were not able to learn the screen-location sequence together with the response-location sequence (one perceptual and one response sequence). In a follow-up experiment, the authors could support these findings by showing that when the participants were instructed to code the response keys in terms of their locations, they did not learn the stimulus-location sequence. By contrast, they did so when they coded their responses in terms of the colors (Gaschler et al., 2012). These findings thus fit the assumption that the implicit learning system can be dedicated to process single features within the visual modality (Hommel, 2009). The stimulus-location sequence and the response-location sequence are both instantiated by the same feature (location) and could not be learned concurrently. In contrast, the stimulus-color and the stimulus-location sequence refer to distinct features (color and location) and could be learned concurrently.

Thus, the empirical findings reported so far suggest that implicit learning, either in the AGL or in the SRTT, relies on associations within features like color, shape, or location as the (minimal) building blocks. However, except for the Conway and Christiansen (2006) study, the experiments all used location as one of the features. This might be a limitation because “location” is suggested to be a special feature (Conway, 2020). First, it seems as if the location of a stimulus is processed more effectively than other perceptual features (Gaschler et al., 2012; Koch & Hoffmann, 2000). Second, location might not be even a pure perceptual feature because it might involve eye movements (Goschke & Bolte, 2012; Marcus et al., 2006; Willingham et al., 2000). To make a stronger point that features might be the (minimal) building blocks of implicit learning, more evidence is needed showing that concurrent learning of two regularities is possible, even when they are instantiated by two non-spatial perceptual features.

## Current study

The current two experiments aimed at testing the hypothesis that implicit learning relies on associations within features like color, shape, or location. In extension to former studies, we tested this for two non-spatial perceptual features (color and shape). An additional purpose was to investigate whether this holds true for the AGL and the SRTT as well. As a starting point, we replicated in Experiment 1 the above mentioned AGL experiment of Conway and Christiansen (2006; Experiment 2a) to explore whether visual-color and visual-shape strings employing two different grammars can be learned concurrently. The purpose of Experiment 2 was then to test the generality of these findings within an SRTT experiment in which we trained the participants concurrently with uncorrelated visual-color and visual-shape sequences.

## Experiment 1

The goal of Experiment 1 was to test whether participants could learn concurrently visual-color and visual-shape strings by replicating the study of Conway and Christiansen (2006, Experiment 2a). In the original study, 20 participants observed alternately short color and shape strings during the familiarization phase. In the test phase, either the acquired color grammar knowledge or the shape grammar knowledge was assessed between participants (10 participants, each). The results showed that the participants in both test conditions had more than chance-level knowledge about either of the two grammars (color sequence Cohen’s *d* > 0.8; shape sequence Cohen’s *d* > 0.5; Cohen, 1988).

## Method

### Participants

An a-priori power analyses (*d* = 0.8,^2^*α* = *β* = 0.05; Faul et al., 2007) yielded a required sample of 40 participants, that is 20 participants^3^ in each condition. We collected the data of 40 participants in an online experiment in Prolific (www.prolific.co; 22 women, mean age = 26.30, age range = 18–40 years, *SD* = 6.08). No participant reported to be color-blind. All participants received £2.50 in exchange for participation.

### Materials

**Fig. 1:**
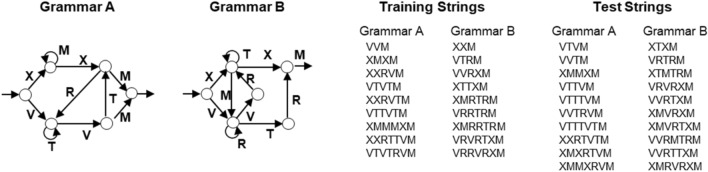
Material from Experiment 1. The illustrations on the left depict Grammar A and Grammar B. From both grammars, nine training strings and ten test strings were generated (on the right). The letters from both grammars were mapped onto colors and shapes. Illustration is based on Conway and Christiansen (2006)

We used the same two different finite-state grammars as Conway and Christiansen (2006) to generate two sets of non-overlapping strings (see Fig. 1). Nine strings of each grammar were used in the training phase and 10 strings of each grammar in the test phase.^4^ The strings contained between three and seven elements. For one grammar, each letter was mapped onto a color vocabulary. For the other grammar, each letter was mapped onto a shape vocabulary. The assignment of vocabulary to grammar was counterbalanced across participants. Also, the assignment of letters to particular colors and shapes was counterbalanced across participants.

We used different sets of colors and shapes than Conway and Christiansen (2006).^5^ For colors, we chose magenta, blue, orange, cyan, and green. For shapes, we presented a triangle, diamond, circle, arch, and cross. All visual stimuli were presented in the center of the screen (80 × 80 pixels).

### Procedure

**Fig. 2:**
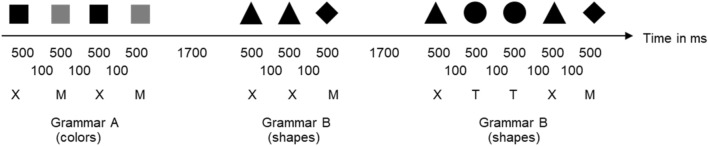
Procedure of Experiment 1. Participants observe each element of the sequences for 500 ms. Elements are separated by 100 ms and sequences by 1.700 ms. Colors (truly colored) and shapes are randomly mapped onto letters. Order of color and shape sequences is completely intermixed. Illustration is based on Conway and Christiansen (2006)

We used the same procedure as in the original experiment. For all participants, the experiment started with computer-presented instructions. Participants were told to observe the shape and color strings carefully, because they would be tested on what they have observed. Then, the training phase started with six training blocks containing the 18 training strings, each. For all participants, half of these strings were derived from one grammar and were presented as colors; the other half relied on the other grammar and was presented as shapes. In each training block, the same 18 training strings were repeated. The presentation order of the strings was entirely random so that the participants observed the grammars intermixed and could learn them simultaneously. Each string element (color or shape item) was presented for 500 ms, followed after an interval of 100 ms by the next string element. Strings were separated by 1.700 ms (see Fig. 2).

After training, the participants were randomly assigned to either the color-test condition or the shape-test condition. In the color-test condition, the participants received only new colored strings derived from either the color grammar (grammatical strings) or the shape grammar (ungrammatical strings) of the training. In the shape-test condition, the participants analogously received only grammatical and ungrammatical shape strings. All participants were informed that the strings they had observed during training were generated according to a complex set of rules. They were told to observe each string carefully and to judge if it follows the same set of rules as in the training phase (grammaticality judgement).

After the test phase, all participants completed an online questionnaire. First, they were asked if they had noticed anything during the experiment. Additionally, the color and shape stimuli were displayed, and participants were asked to indicate with which color and shape the strings usually began and ended. They were also asked to produce as many transitions as they believed had occurred in the training stimuli. For each correct begin, ending, and transition, the participants gained a point so that a score of explicit grammar knowledge could be calculated.

### Data analysis

To assess the participants’ grammar knowledge from the test phase, we applied the same scoring system as in the original analysis (Conway & Christiansen, 2006). The participants’ judgments were scored as correct, when they classified a test string as grammatical that has been derived from that particular grammar during training (e.g., a color-test string was classified as grammatical, and the test string was based on the color grammar). If they classified a test string as ungrammatical and it was based on the other grammar than during training, the judgment was classified also as correct (e.g., a color-test string was classified as ungrammatical, and the test string was derived from the shape grammar). If the participants had learned the transitions between the respective features, they should be better than chance to correctly accept or reject the test strings as grammatical or ungrammatical (> 50% correct judgements). It is important to note that chance-level performance would lead to two possible interpretations. The first interpretation would be that participants did not learn the transitions. The second interpretation might be that the participants acquired abstract representations of both grammars without distinguishing between the perceptual characteristics. In this case, for instance, participants would judge most of the strings as grammatical because all strings are either based on the shape grammar or the color grammar. Due to the scoring system, their judgements would lead to chance-level performance. Hence, the scoring system cannot differentiate between a participant who did not learn at all and a participant who learned abstract grammar rules. However, a scoring system above chance-level would clearly identify a participant who learned both grammars separately (Conway & Christiansen, 2006).

We first report the frequentist analyses and afterwards additionally the Bayes factors. For all Bayes analyses, we tested one-sided hypotheses and used a default Cauchy prior distribution with *r* = 1/√2, truncated to only allow positive effect sizes (van Doorn et al., 2019). All Bayes analyses were conducted with JASP (2020) (Wagenmakers et al., 2018).

## Results and discussion

Table 1 reports how many of the 20 test strings were classified correctly. The first line describes the performance of the whole sample, and the other lines allow the comparison of the performances in the color-test and the shape-test condition. An asterisk marks whether the percentages were above chance-level (50%).

**Table 1:** Results of Experiment 1

Condition	Number correct	Percentage correct	*n*
Both conditions	11.97 (2.80)	60 (13.98)***	40
Color-test condition	12.40 (2.66)	62 (13.32)***	20
Shape-test condition	11.55 (2.93)	58 (14.64)*	20

The table shows the mean number, mean percent correct responses, standard deviations (in parentheses), and results of tests of significance (versus chance) in the whole sample and in the two conditions of Experiment 1

The number correct is out of a possible maximum of 20

All *t* tests were one-tailed

*p < .05

**p < .01

***p < .001

The results in Table 1 show that the participants in the color-test condition were better able than chance to correctly classify the test strings (*t*(19) = 4.03, *p* < .001, *d* = 0.90 [0.37–1.42], ${\mathrm{BF}}_{10}$ = 97.79 [85.92–98.28]). The Bayes factor indicated very strong evidence for the hypothesis that the color-grammar had been learned.

The participants in the shape-test condition classified the test strings better than chance-level (*t*(19) = 2.37, *p* = .014, *d* = 0.53 [0.05–0.99], ${\mathrm{BF}}_{10}$ = 4.24 [2.85–4.76]). The Bayes factors indicated moderate evidence for the hypothesis that the shape-grammar had been learned.

**Fig. 3:**
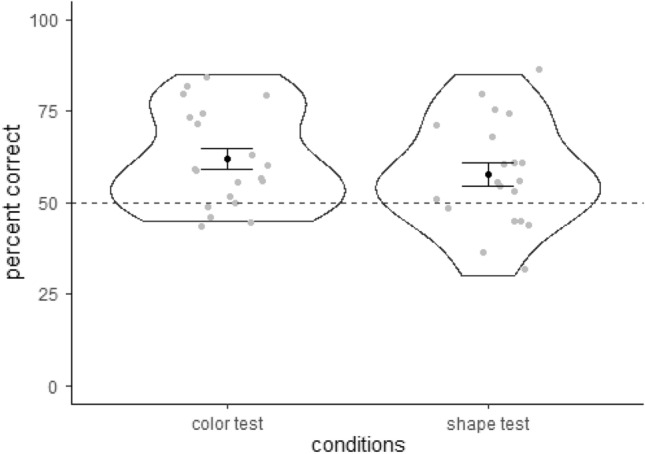
Percent Correct Responses as a Function of Condition in Experiment 1. Grey circles represent percent correct responses for each participant. Black circles depict observed means. Error bars are standard errors of means. Chance-level was 50% and is indicated by the dashed grey line. The figure was created with R (2020)

Jittered violin plots depicting the percent correct responses for each individual participant in the two conditions are provided in Appendix A, Fig. 3. The plots show that most of the participants in both conditions correctly responded above chance-level.

The analyses of the explicit knowledge indicated that the number of the verbally named transitions between the participants in the color-test versus the shape-test conditions did not differ (color-test condition: *M* = 2.45, *SD* = 1.43; shape-test condition: *M* = 2.15, *SD* = 1.14; *t*(38) = 0.73, *p* = .468, ${\mathrm{BF}}_{10}$ = 0.38 [0.22–1]). Additionally, we calculated the correlation between the participants’ performance in the test phase and the number of the verbally named transitions (color-test condition: *r* = 0.11, *t*(18) = 0.50, *p* = .627, ${\mathrm{BF}}_{10}$ = 0.31 [− 0.32–0.50]); shape-test condition: *r* =  − 0.03, *t*(18) =  − 0.11, *p* = .913, ${\mathrm{BF}}_{10}$ = 0.28 [− 0.43–0.39]). Thus, it seems as if the participants’ grammaticality judgments were mainly driven by implicit grammar knowledge.

In conclusion, our results replicated the findings of Conway and Christiansen (2006) that the participants were able to learn a color- and a shape grammar concurrently. However, the evidence for the learning of the color grammar was numerically stronger than that for the shape grammar. Therefore, an alternative explanation could be that it is more difficult to simultaneously learn color and shape strings because these features are both perceptual and as such the participants do not learn the two underlying grammars entirely independent from each other (Abrahamse et al., 2010). As a second alternative explanation, it is conceivable that the learning of the color and shape strings interfered with each other because the color strings were displayed in colored squares. We will discuss these alternative explanations more thoroughly in the General Discussion section. In Experiment 2, we used the same stimuli but a different method to test whether these findings are also valid for the SRTT paradigm and to possibly clarify these two alternative accounts.

## Experiment 2

Experiment 2 aimed at testing once again that implicit learning relies on associations within features like color and shape. We tested whether the participants could learn concurrently a visual-color and a visual-shape sequence, but this time within an adapted SRTT paradigm of Eberhardt and colleagues (2017). During learning, the participants observed two uncorrelated visual-color and visual-shape sequences. After the training phase, we assessed the participants’ knowledge about either the color or the shape sequence in a post decision wagering task (again between participants).

We decided to assess the participants’ sequence knowledge in an off-line test, a post decision wagering task (Persaud et al., 2007) because usually learning of a pure perceptual sequence leads to rather small learning effects (Haider et al., 2012) A learned pure perceptual sequence accelerates only the encoding processes while the response selection process does not profit from the implemented sequence. Thus, the potential contribution to faster response times is much smaller in perceptual than in response-based sequence learning (Haider et al., 2012).

If the implicit learning system relied on the separate processing of modalities (e.g., vision, hearing, etc.) and all their related feature information, then a visual-color and a visual-shape sequence would both be processed within the same module which should make parallel learning more difficult or even impossible. By contrast, if single features within the same modality, such as color and shape, are processed in distinct modules in the implicit learning system, the participants should be able to learn both sequences concurrently. Thus, if participants showed above chance-level knowledge in our post decision wagering task for both sequences (i.e., in both conditions), this would denote that color and shape are processed separately in the implicit learning system (Hommel, 2009). This would strengthen the assumption that implicit sequence learning relies on associations within features, even when these features are not spatial (Eberhardt et al., 2017; Haider et al., 2018; Hommel et al., 2001).

## Method

### Participants

An a-priori power analyses (*d* = 0.6,^6^*α* = *β* = 0.05; Faul et al., 2007) yielded a required sample of 60 participants, that is 30 participants in each condition. Participants were excluded if they had made more than 20% errors in the training or test phase. No participant reported to be color-blind.

Sixty-four students of the University of Cologne participated in the laboratory experiment. No participant exceeded our error criterion. One participant in the shape-test condition and one participant in the color-test condition were excluded due to technical issues. This left 30 participants in the color-test condition and 32 participants in the shape-test condition (45 women, 1 not stated, mean age = 22.98, age range = 18–38 years, SD = 5.04). All participants received either 4€ or course credit in exchange for participation. In addition, they could earn 2€ extra money in the wagering task.

### Material

In the training phase, in every trial two targets appeared in the middle of the screen (100 × 100 pixels, 2 cm distance) in front of a grey background. The color-target always occurred on the left side, the shape-target on the right. Unbeknownst to the participants, either the colors followed a 6-element first-order sequence and the shapes a 7-element second-order sequence (magenta – blue – orange – cyan – green – red; diamond – cross – circle – arch – triangle – cross – star) or vice versa (blue – magenta – green – orange – red – magenta – cyan; cross – diamond – arch – star – circle – triangle). Due to the differences in sequence length, the correlated sequence was rather long (42 elements), making it highly unlikely that the participants would learn this long sequence (e.g., Schmidtke & Heuer, 1997). To ensure that the participants would attend to the colors and the shapes, we interspersed some differing trials. In 16.7% of the trials the color target was dotted and in another 16.7% of the trials the frame of the shape target was dashed. Participants had to indicate these differing targets by pressing the spacebar.

### Procedure

The experiment started with instructions displayed on the screen. The participants were told to pay close attention to the shape and the color-patch stimuli and to press the spacebar as soon as they detect a differing target. Afterwards, the training phase started with 7 training blocks containing 89 trials, each. In each trial, a color and a shape stimulus appeared on the screen for 250 ms. After an inter-trial-interval of 2150 ms the next targets appeared on the screen. The participants merely observed the color and shape targets. In some trials, either the color target was dotted, or the shape target was dashed. If this was the case, the participants had to press the spacebar within the inter-trial-interval of 2150 ms. If they missed a differing target, an error message (“Miss”) appeared for 250 ms and a 400 Hz tone sounded for 50 ms. If they pressed the spacebar, but both targets did not differ, the message “False Alarm!” appeared on the screen for 250 ms and the 400 Hz tone also sounded for 50 ms. Between blocks, the participants could take short breaks.

**Fig. 4:**
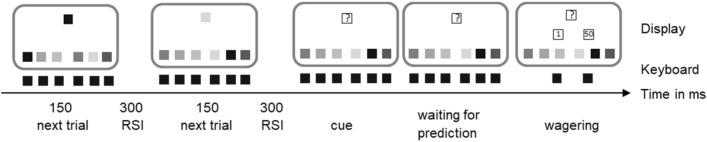
Three Trials of the Color-Test Phase in Experiment 2. On the left, two regular trials are depicted. Six response squares occur at the bottom of the screen (truly colored). Response keys are mapped to the keys Y, X, C, B, N, and M on a QWERTZ keyboard. In the upper part of the screen, a target color appears. The target disappears after 150 ms and the response squares remain on the screen until the participants’ answer. Participants are supposed to find the same color among the response squares and press the corresponding key. In the first trial, the left-most key Y needs to be pressed. After the participants’ key press, the screen is blank for 300 ms. After that, the next trial starts. Here, the next target color according to the color sequence is displayed. The arrangement of the keys changes from trial to trial to impede a motor sequence of key presses. On the right, a wagering trial is depicted. In the upper part of the screen, instead of the next target color, a question mark appears. Participants are supposed to guess the next color by pressing the corresponding key. After their guess, two cent-amounts appear on the screen indicating the wager options. After the participant chooses one of the wagers by pressing the corresponding key (A or K) and a response–stimulus interval (RSI) of 300 ms, the next non-wager trial starts with a new arrangement of response squares

After the training phase, the participants were administered to the post decision wagering task (see Fig. 4) which served to assess the participants knowledge about either the color or the shape sequence. Accordingly, the participants were randomly assigned to either the color-test or the shape-test conditions.

The post decision wagering task consisted of 100 trials presented within one single block. In these trials, a single target (either a color patch in the color-test condition or a shape in the shape-test condition) appeared in the upper part of the screen. In the lower part of the screen, six colored response squares (color-test condition) or six shapes (shape-test condition) appeared. Their screen locations were mapped to the keys Y, X, C, B, N, and M on a German QWERTZ keyboard (the left most response square was mapped to the Y-key, etc.). The participant’s task was to press the response key assigned to the location of the response square containing the target. The arrangement of the colors (shapes) of the response squares changed from trial to trial such that the response keys did not follow any sequence.

While the target disappeared after 150 ms, the response squares remained on the screen until the participant`s response. Afterwards, the screen went black for 300 ms. Incorrect responses were not signaled.

In overall 20 trials, the so-called wagering trials, a question mark appeared instead of the target. The participants’ task here was to guess the color or shape of the current target. That is, they had to search among the six response squares the color or shape (depending on condition) they guessed the target would have been and to press the respective response key. Immediately after the participant’s response, the pictures of a 1 cent and a 50 cents coin appeared on the screen. The participants were instructed to bet on the correctness of their guessed target by pressing either the A-key (1 cent) or the K-key (50 cents). If their guess was correct, they won the amount of the wager; if not, they lost it. Importantly, the participants were not informed whether they guessed correctly in the respective trials. The participants were free to use low and high wagers as frequently as they were pleased to, but they were told to try to maximize their earnings.

The following example serves to illustrate the wagering phase further: A participant who observed the 6-element color sequence in the training phase (magenta – blue – orange – cyan – green – red) would react by simple key presses first to the color magenta and then in the next trial to blue. After that, instead of the next color orange, a question mark appears, and the participant is supposed to guess that the color orange would have followed in the training phase.

In the wagering trials, the arrangement of the response squares did not change from trial t-1 to t. The participants might remember the location of their last response square (but not the last color or shape) and attend to this position in order to predict the next target. If this were the case, changing the arrangement of the response squares would overwrite the representation of the last target (see Fig. 4). The maximum of extra-earnings was set to 2€. If the participants reached this maximum before the end of the block, the wagering task was terminated.

After having finished the post decision wagering task, a short interview followed to assess the participants’ reportable sequence knowledge. The participants were first asked if they had noticed anything during the experiment. Then, they were asked if they had noticed any structural regularity during their experiment and, if they affirmed, they were asked to describe it. Subsequently, they were informed about the two sequences and their lengths and asked to try to reconstruct both, the color and the shape sequences. Participants were categorized as having structural conscious knowledge when they were able to name three or more consecutive transitions of their test sequence. Finally, participants received their payment or course credit, were informed about their earnings in the wagering task and were then debriefed.

### Data analysis

If the participants had learned the sequences, their correct guesses in the wagering task should be above chance-level.^7^ We set the chance-level to 20% since immediate repetitions of colors or shapes in the respective sequences were excluded during training.^8^ In order to assess whether the participants’ sequence knowledge was implicit or explicit, we also analyzed the percentage of correct answers under the condition of high wagers versus of low wagers. The rationale was that if their correct guesses were mainly driven by implicit sequence knowledge, they will place high or low wagers randomly and will be unable to maximize their overall earnings (Dienes & Seth, 2010). If, by contrast, the participants have acquired explicit sequence knowledge, they should be able to strategically place high wagers when they are certain that their guess of the last target-color or target-shape was correct (see Haider et al., 2011).

Again, we report results from frequentist analyses and Bayes factor analyses using JASP (2020), (Wagenmakers et al., 2018) with default Cauchy prior distribution with *r* = 1/√2 allowing only positive effect sizes (van Doorn et al., 2019).

## Results and discussion

Table 2 shows the error and misses rates during the training phase separately for the colors and shapes and the color-test and the shape-test conditions. The error rates include the misses and false alarms for the color and shape stimuli. Additionally, we compared the miss rates between the two conditions and between the stimuli to ensure the participants were equally attentive to the color- and shape-sequences. As can be seen, the error rates and misses did not differ between the color-test and shape-test condition (error rates: *t*(60) =  − 0.84, *p* = .403, ${\mathrm{BF}}_{10}$ = 0.35 [0.2–1]; misses color: *t*(60) =  − 1.19, *p* = .238, ${\mathrm{BF}}_{10}$ = 0.47 [0.27–1]; misses shape: *t*(60) =  − 0.40, *p* = .691, ${\mathrm{BF}}_{10}$ = 0.28 [0.15–1]). Hence, the participants were equally attentive to the color and shape stimuli in both conditions. Overall, the participants of both test conditions did miss more colors than shapes (*t*(61) = 3.71, *p* < .001, ${\mathrm{BF}}_{10}$ = 55.08 [35.56–61.84]), indicating that the dotted colors were slightly harder to detect than the dashed shapes.

**Table 2:** Error rates of the training phase in experiment 2

Condition	Errors	Misses color	Misses shape	*n*
Both conditions	1.10 (1.14)	2.50 (4.30)	0.67 (1.10)	62
Color-test condition	0.97 (0.90)	1.83 (2.50)	0.61 (1.03)	30
Shape-test condition	1.21 (1.34)	3.12 (5.45)	0.72 (1.17)	32

The table shows the overall mean percent errors, misses of dotted colors and misses of dashed shapes, and standard deviations (in parentheses) as well as for each condition of Experiment 2

Table 3 shows the findings of the wagering task. The columns present the overall mean percent of correct responses as well as separately the mean percent correct responses under the condition of high versus low wagers.

**Table 3:** Results of the post decision wagering task in Experiment 2

Condition	Percent correct	Correct | high wager	Correct | low wager	*n*
Both conditions	26.53 (16.93)**	26.69 (21.82)	27.65 (24.17)	62
Color-test condition	26.00 (16.78)*	25.25 (22.22)	25.45 (22.58)	30
Shape-test condition	27.03 (17.31)*	28.06 (21.81)	29.76 (25.91)	32

The table shows the overall mean percent correct responses and standard deviations (in parentheses) as well as percent correct responses when subjects placed high or low wagers in general and in the two conditions of Experiment 2

The chance-level was 20%

All *t* tests were one-tailed

*p < .05

**p < .01

***p < .001

As can be seen from Table 3, the participants of the entire sample had more sequence knowledge than expected by chance (chance-level was 20%), *t*(61) = 3.04, *p* = .002, *d* = 0.38 [0.13–0.64], ${\mathrm{BF}}_{10}$ = 17.31 [10.45–21.86]). This significant learning effect was also found when testing separately for the color- and the shape-test condition (color sequence: *t*(29) = 1.96, *p* = .030, *d* = 0.36 [− 0.01–0.72], ${\mathrm{BF}}_{10}$ = 2.00 [1.19–2.80]; shape sequence: *t*(31) = 2.30, *p* = .014, *d* = 0.41 [0.04–0.76], ${\mathrm{BF}}_{10}$ = 3.61 [2.22–4.60]). However, while the Bayes analyses only suggested anecdotal evidence for the color-learning effect it was moderate for the shape-learning effect.

**Fig. 5:**
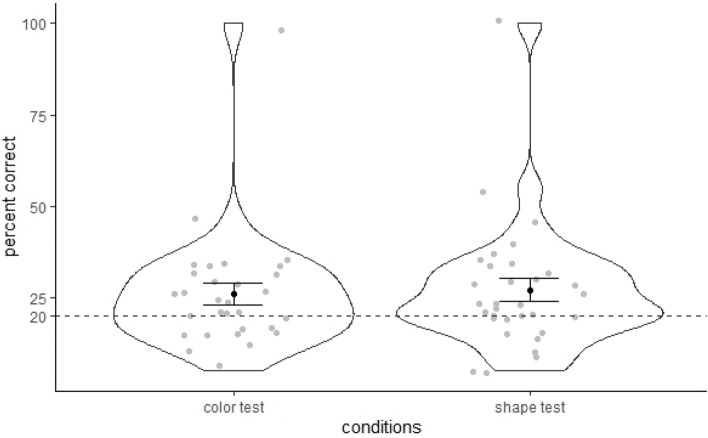
Percent Correct Responses as a Function of Condition in Experiment 2. Grey circles represent percent correct responses for each participant. Black circles depict observed means. Error bars are standard errors of means. Chance-level was 20% and is indicated by the dashed gray line. The figure was created with R (2020)

Jittered violin plots depicting the percent correct responses for each individual participant in the two conditions are provided in Appendix B, Fig. 5. The plots show that most of the participants in both conditions showed a learning effect, indicating that our measures of the central tendencies are in line with the data.

In order to test whether the knowledge was implicit or explicit, we additionally analyzed the percent correct responses under the conditions of high versus low wagers. The results are also shown in Table 3. In both test-conditions, the percent correct responses when participants placed high wagers did not differ from the percent correct responses when they placed low wagers (color-test condition: *t*(22) =  − 0.04, *p* = .517, ${\mathrm{BF}}_{10}$ = 0.21 [0.11–1]; shape-test condition: *t*(23) =  − 0.33, *p* = .627, ${\mathrm{BF}}_{10}$ = 1.17 [0.09–1]). Thus, in the color-test condition, there is moderate evidence that the sequence knowledge was rather implicit than explicit. In the shape-test condition, the evidence for the implicit nature of the sequence knowledge was only anecdotal.

Nevertheless, one participant in each of the two test-conditions reached the maximum of earnings in the wagering task. If these two participants with explicit sequence knowledge were excluded from the implicit knowledge analysis, the frequentist *t* tests still remained significant (all *p*’s < 0.03).

To summarize, the participants in both conditions showed sequence learning effects. The additional analyses suggest that this learning effects were rather due to implicit than to explicit knowledge. Since all participants were randomly assigned to the two test conditions, the conclusion seems justified that the participants learned both sequences concurrently. This supports our assumption that colors and shapes refer to distinct features separately represented in the implicit learning system. However, as in Experiment 1, the participants showed stronger learning effects for one perceptual feature than for the other (stronger learning effects for color strings in Experiment 1 and for the shape sequence in Experiment 2).

## General discussion

The reported experiments provided two main results. Experiment 1 replicated the findings of Conway and Christiansen (2006) showing that the participants can learn two grammars concurrently when one is instantiated by color strings and the other by shape strings. In Experiment 2, we generalized these findings to the SRTT by showing that the participants can learn a color sequence together with a shape sequence. Thus, the two experiments converge to the same conclusion that implicit learning is based on associations within basic features. Nevertheless, a few methodological issues need some discussion.

The first point concerns the material used in our replication of the Conway and Christiansen (2006) study. In the original study, the authors used hard to verbalize colors and shapes to impede a verbal coding strategy. Since we could not, in a first attempt, replicate their findings with slightly different shades of colors (red shades instead of blue and green shades), we used easy to pronounce colors (red, blue etc.) in the here reported experiments. This might have fostered the acquisition of explicit representations about the strings. However, the findings of Experiment 2, in which we tested for explicit knowledge, revealed that, at least in the SRTT, the participants’ knowledge about the sequence was implicit rather than explicit. The Bayes factors indicated moderate evidence for the implicit nature of the knowledge about the color sequence and anecdotal evidence for the shape sequence. Thus, the acquisition of some explicit knowledge cannot be ruled out entirely, but it is an open question whether using the material of Conway and Christiansen (2006) would have led to different results. For future studies, it might be worthwhile to carefully assess the amount of explicit knowledge to ensure the significance of the results for the implicit learning system.

The just mentioned unsuccessful replication of the findings of Conway and Christiansen (2006) was unexpected since other researchers were already able to show concurrent color and shape learning in the AGL (Johansson, 2009; Turk-Browne et al., 2008; Walk & Conway, 2016). Yet, this discrepancy between former and our findings suggests that the concurrent learning of two different feature regularities within the visual modality might be rather fragile. Already small differences in the difficulty to process the colors and shapes might be rather critical for the learning of both regularities. If one of the two features is harder to process, it probably receives more attention resulting in smaller learning effects for the respective other feature (Memelink & Hommel, 2012). In a similar vein, for instance, Deroost and Soetens (2006) could not replicate within an SRTT the findings of Mayr (1996) after having changed the presentation format of the stimuli. Moreover, in our experiments, the learning effects differed between the features. In Experiment 1, the participants showed only moderate evidence for learning of the shape strings and in Experiment 2, they showed only anecdotal evidence for color sequence learning. A possible explanation might be that we displayed the color regularities in colored squares in both experiments. Hence, there could have been some amount of interference between the pure shape sequence and the color sequence that was displayed by shapes. Overall, these observations suggest that slight differences in the experimental setups seem to be sufficient to not finding concurrent implicit learning of two regularities. This might be due to implicit learning depending on selective attention (Chun & Turk-Browne, 2008; Jiang & Chun, 2001; Jiménez & Méndez, 1999).

A second limitation of our study is that we did not assess concurrent learning of two perceptual sequences within participants. All participants observed both regularities during the training phase, but afterwards were tested for only their knowledge about one of the two regularities. Therefore, an alternative explanation for our results might be that the participants paid more attention to either the regularity of the color or of the shape stimuli and hence, learned only the attended sequence, and not both.

For the SRTT, we already reported that the number of misses in the training phase did not differ between the two features suggesting that the participants had been equally attentive to colors and shapes. To further test for this limitation, we correlated the number of misses for color and shape stimuli during the training phase separately for the two test-conditions. If participants focused their attention to only one feature, let us say colors, they should have missed more dotted shapes, indicated by a negative correlation between the misses of colors and shapes. However, for both test conditions of Experiment 2, this correlation was positive rather than negative (color-test condition: *r* = 0.31, *t*(28) = 1.72, *p* = .096, ${\mathrm{BF}}_{10}$ = 0.85; shape-test condition: *r* = 0.59, *t*(30) = 3.99, *p* < .001, ${\mathrm{BF}}_{10}$ = 89.03). Thus, the participants did not unequally distribute their attention towards either the shapes or the colors during the training phase.

**Fig. 6:**
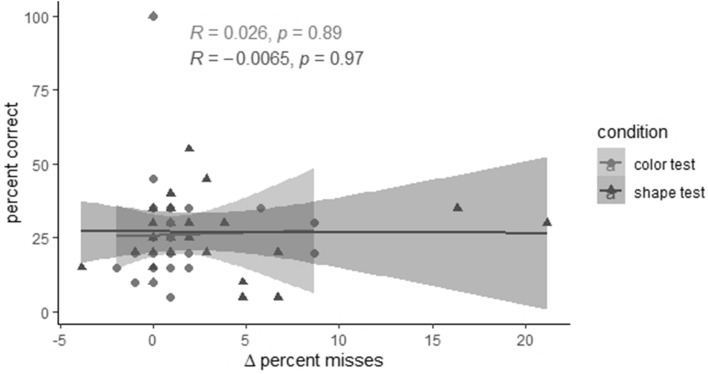
Percent Correct Responses as a Function of the Difference between Percent Misses of deviant Color and Shape Stimuli in Experiment 2. On the X-axis, the difference between the misses of dotted color and dashed shape stimuli in percent is depicted (percent color misses–percent shape misses). On the Y-axis, the percent correct responses in the post decision wagering task are depicted. Light-grey circles represent the participants in the color-test condition. Dark-grey triangles represent the participants in the shape-test condition. The light-grey and dark-grey areas depict the 95% confidence interval of the regression lines of the respective conditions. The graph shows that the participants did not pay systematically more attention in the test phase to the feature for that they show a learning effect in the test phase. The figure was created with R (2020)

Furthermore, if the participants paid more attention to one of the two stimulus features, the miss rate during the training phase should have been higher for one of these features, on the one hand, and, on the other hand, the amount of knowledge for this stimulus feature should have been lower in the post decision wagering task. For this purpose, we correlated the difference between the two miss rates (color misses – shape misses) during the training phase separately with the percent correct responses in either the color or the shape test. The correlations were both insignificant (color-test condition: *r* = 0.03, *t*(28) = 0.14, *p* = .89, ${\mathrm{BF}}_{10}$ = 0.23; shape-test condition: *r* =  − 0.006, *t*(30) =  − 0.04, *p* = .972, ${\mathrm{BF}}_{10}$ = 0.22; see Appendix B, Fig. 6 for a graphical overview). This finding thus runs again counter to the argument that the participants distributed their attention differently to the color or the shape sequences during the SRTT training phase. Rather, it suggests that the participants attended to both sequences to the same extent. Nonetheless, testing concurrent learning of two perceptual sequences within participants would provide even stronger evidence for the hypothesis that associations within features (e.g., color, shape, location, etc.) might be the foundation of implicit learning (Eberhardt et al., 2017; Haider et al., 2018; Hommel et al., 2001). This seems to be true for implicit learning within the SRTT and the AGL as well.

For the broader theoretical perspective, our results contribute to a clarification of modularized theories of implicit learning. As described in the Introduction, Keele and colleagues (2003) stated in their Dual-System Model that multiple regularities can be learned concurrently as long as they are processed in distinct encapsulated modules. The respective dimension serves as the selection criteria for what is processed in a single module (Keele et al., 2003). However, the authors acknowledged already that research is needed to better define the term dimension. Previous research, so far, proposed that a dimension should be equated with modality (Abrahamse et al., 2010). Our results suggest that Keele’s term dimension can better be equated with the concept of features in the sense of Hommel (2009) since we found concurrent learning of two perceptual regularities that differed only with regard to their instantiated features color or shape. This is also in line with the assumptions of Frost et al., (2015) who argue that multiple regularities can be learned concurrently as long as they are processed in distinct neuronal networks. The stimulus domain influences what is processed in a single neuronal network. Our results suggest that the term domain can also be equated with the term feature.

On a more methodological perspective, our results suggest that implicit learning in the AGL and the SRTT might rely on the same mechanisms. Thus, our findings might help to better connect these two rather separated research fields (for a deeper comparison see Christiansen, 2019; and Frost et al., 2015).^9^

A last important point is that we do not want to exclude that implicit learning could rely on associations between more complex stimuli. It is entirely conceivable that participants when confronted with contingencies between more complex stimuli, like for instance, categories (Goschke & Bolte, 2012), also will learn these associations implicitly. We also do not challenge the evidence of cross-modal learning in which dependencies between two features are learned (e.g., a multimodal sequence of auditory and visual words; Kemeny & Meier, 2016; Seitz et al., 2007; Thiessen, 2010; see Conway, 2020, for a detailed summary of this line of research). For instance, Keele et al., (2003) argue that such cross-modal learning is based on the multimodal system but depends on selective attention. Frost et al., (2015) would also admit the possibility for cross-modal learning given the complexity and similarity of the stimuli. However, both models do not further specify the constraints needed for implicit cross-modal learning to occur. In addition, on the empirical side some findings suggest that cross-modal implicit learning seems to be difficult to find (e.g., no learning of a visual color-shape sequence; Walk & Conway, 2016). Hence, an interesting question for further research is to investigate whether across-feature integration in implicit learning depends on the same constraints as implicit learning within one single feature. For instance, it is unclear whether learned associations between complex stimuli have a higher probability to become consciously accessible than those within a feature (Conway, 2020; Keele et al., 2003).

Nonetheless, the different magnitudes of the color and shape learning effects suggest that human behavior does not just surrender to the perceptual input from the environment. In implicit learning, only the acquired associations between the features of the target stimuli are unconscious, but implicit learning requires the conscious processing of the target stimuli (Baars, 2002). This processing of the target stimuli depends on bottom-up saliences and top-down intentional weighting so that characteristics of the targets influence the processing of its features and hence, on their impact on human behavior (Cleeremans et al., 2020; Memelink & Hommel, 2012). Future research should address how far the top-down factors, such as the task set, influence the mechanisms of implicit perceptual learning.

To conclude, the aim of our study was to contribute to the question about the building blocks of implicit learning. Previous research has shown that implicit learning can be modality-specific (e.g., Deroost & Soetens, 2006; Mayr, 1996) and under specific circumstances even cross-modal (see Conway, 2020 for an overview). In line with the findings of Conway and Christiansen (2006) and Eberhardt et al., (2017) our study adds to that and clearly suggest that at least when participants had to learn contingencies between simple stimuli, implicit learning is based on associations within features (Hommel et al., 2001).

### Electronic supplementary material

Below is the link to the electronic supplementary material.

Supplementary file1 (XLSX 81 KB)

Supplementary file2 (PDF 112 KB)

Supplementary file3 (XLSX 540 KB)

Supplementary file4 (XLSX 2510 KB)

Supplementary file5 (PDF 92 KB)

Supplementary file6 (PDF 90 KB)

## Data Availability

The data sets and analysis scripts for R are available online (https://osf.io/fc7wv/?view_only=0104c92fa72d46928f652a221675a376).

## Appendix A

Additional analyses for Experiment 1

## Appendix B

Additional analyses for Experiment 2

## Footnotes

1 In the description of the Dual-System Model, we mainly focused on the encapsulated modules of the unidimensional system. However, Keele and colleagues (2003) also postulated a multidimensional system that is thought to integrate information across different dimensions. The only constraint here is that the information to be associated is attended. Thus, learning relies on attentional resources. As a consequence, the resulting knowledge is, at least in principle, accessible to consciousness.

2 Conway & Christiansen (2006) reported an effect size of *d* = 0.8 in their experiment.

3 Johansson (2009) also reported a sample size of 20 participants in each condition in their replication.

4 We replaced one string of Conway & Christiansen (2006) that was reported twice in their material with a string used by Johansson (2009).

5 Conway and Christiansen (2006) used in their original study a different set of stimuli, namely hard-to-verbalize shapes and colors ranging along a continuum from light blue to green to prevent a verbal coding strategy. In a pilot study, we also used hard-to-verbalize shapes and colors. While we used the same shapes as Conway and Christiansen (2006), our colors ranged along a continuum from light red to dark red. However, participants in this pilot study learned only the color grammar. We discuss how the perceptual distinctness might affect learning in the General Discussion section. Regarding our Experiments, it should be noted that we used colors and shapes that could be labelled.

6 The effect size d = 0.6 is derived from the smallest effects in Eberhardt and colleagues (2017).

7 We did not report any response times of the test phase. Unlike the standard SRTT, all trials followed a sequence. The sequence was not replaced by a random sequence. Hence, a comparison of response times would not indicate whether the sequence has been learned.

8 The participants guessed the correct answer out of six targets. However, since they never experienced a direct repetition of a target, they could exclude the target from trial t-1 and hence, chance-level was 1 out of 5 targets (20%).

9 It should be noted that the training and test phases of the AGL and SRTT differ, and more research is needed to test whether learning within these two paradigms relies on the same mechanisms. However, our research indicates, at least, that the smallest entities that can become associated in the AGL and SRTT are the same, namely features.
