# Supplementary material for: Concurrent visual sequence learning
Source: Psychol Res. 2023 Mar 22;87(7):2086–100. doi: 10.1007/s00426-023-01810-2 (PMC10457409; doi:10.1007/s00426-023-01810-2)
Supplement: Supplementary file 2 — Supplementary file2 (PDF 112 KB) [file 426_2023_1810_MOESM2_ESM.pdf]

Experiment 1 Data Legend

This document belongs to the article "Concurrent Visual Sequence Learning" by Sarah Wilts and Hilde Haider (sarah.wilts@uni-koeln.de, University of Cologne) submitted to the journal Psychological Research.

In this document the variable names and levels of the Excel file „Experiment1_data set" are explained. The Excel file contains the raw data set of the test phase of the artificial grammar learning experiment.

subject_number	The numbers of the subjects from 1 to 44 including the participants 14 and 16 that were distracted during the experiment and therefore, excluded from data analyses.
trainmat	In condition 1, the color vocabulary was assigned to the grammar 1 and the shape vocabulary to grammar 2.<br>In condition 2, the shape vocabulary was assigned to grammar 1 and the color vocabulary to grammar 2.
testmat	In condition 1, the participants conducted a color-test.<br>In condition 2, the participants conducted a shape-test.
trial	The test phase contained 20 trials.
grammar	The test string was either derived from grammar 1 or grammar 2.
string	This column indicates the number of the exact string that was tested in the respective trial. In total, 20 strings were tested in random order.
position1 to position7	These columns present the single elements (colors or shapes) of the test strings. The strings contained between three and seven elements. The numbers 1 to 5 indicate which element was displayed. A zero indicates that the string was already finished.
response	The participants indicated whether the sequence was grammatical (yes = 1) or ungrammatical (no = 0).

response_time	The computer measured the response time in seconds.
color_hit	The participants‘ judgments were scored as correct (1), when they classified a color test string as grammatical that was derived from the color-grammar.
color_ correctrejection	The participants‘ judgments were also scored as correct (1), when they classified a color test string as ungrammatical that was derived from the shape-grammar.
shape_hit	The participants‘ judgments were scored as correct (1), when they classified a shape test string as grammatical that was derived from the shape-grammar.
shape_ correctrejection	The participants‘ judgments were also scored as correct (1), when they classified a shape test string as ungrammatical that was derived from the color-grammar.
explicit_ color	The participants' score of explicit color knowledge. Higher scores indicate higher amounts of explicit color knowledge.
explicit_shape	The participants' score of explicit shape knowledge. Higher scores indicate higher amounts of explicit shape knowledge.
