# Supplementary material for: Concurrent visual sequence learning
Source: Psychol Res. 2023 Mar 22;87(7):2086–100. doi: 10.1007/s00426-023-01810-2 (PMC10457409; doi:10.1007/s00426-023-01810-2)
Supplement: Supplementary file 5 — Supplementary file5 (PDF 92 KB) [file 426_2023_1810_MOESM5_ESM.pdf]

Experiment 2 – Test – Data Legend

This document belongs to the article "Concurrent Visual Sequence Learning" by Sarah Wilts and Hilde Haider (sarah.wilts@uni-koeln.de, University of Cologne) submitted to the journal Psychological Research.

In this document the variable names and levels of the Excel file „Experiment2_test_data set" are explained. The Excel file contains the raw data set of the test phase of the serial reaction time task: the post decision wagering task.

subject_number	The numbers of the subjects from 1 to 64. The data of the participants 48 and 58 were not recorded due to technical issues.
condition	The participants were randomly assigned to the color-test condition (1) or the shape-test condition (2).
trial	The post decision wagering task contained 100 trials.
Y, X, C, B, N, M	These columns indicate which colors in the color-test condition or shapes in the shape-test condition were assigned to the keys Y, X, C, B, N, and M on a QWERTZ keyboard (color-test condition: 1 = red, 2 = blue, 3 = green, 4 = orange, 5 = magenta, 6 = cyan; shape-test condition: 1 = triangle, 2 = diamond, 3 = circle, 4 = arch, 5 = cross, 6 = star)
color target	If the participant was in the color-test condition, the color of the target in this trial is indicated (1 = red, 2 = blue, 3 = green, 4 = orange, 5 = magenta, 6 = cyan). Otherwise, a zero is displayed.
shape target	If the participant was in the shape-test condition, the shape of the target in this trial is indicated (1 = triangle, 2 = diamond, 3 = circle, 4 = arch, 5 = cross, 6 = star). Otherwise, a zero is displayed.
amot	This column indicates the correct response key (1 = Y, 2 = X, 3 = C, 4 = B, 5 = N, 6 = M).

antw	This column indicates the actual response key presses of the participants (1 = Y, 2 = X, 3 = C, 4 = B, 5 = N, 6 = M).
error	In this column, an erroneous trial is indicated (1).
trial_after_error	Since also the trials after an erroneous trial were excluded from the main analyses, these trials are also marked (1).
wagering_trial	In this column, the twenty wagering trials are indicated (1).
wager	The participants either bet 0,01€ or 0,50€ on their guess.
profit	This column indicates the current profit in this trial.
response_time	The computer measured the response times in milliseconds.
