# Supplementary material for: Concurrent visual sequence learning
Source: Psychol Res. 2023 Mar 22;87(7):2086–100. doi: 10.1007/s00426-023-01810-2 (PMC10457409; doi:10.1007/s00426-023-01810-2)
Supplement: Supplementary file 6 — Supplementary file6 (PDF 90 KB) [file 426_2023_1810_MOESM6_ESM.pdf]

Experiment 2 – Training – Data Legend

This document belongs to the article “Concurrent Visual Sequence Learning” by Sarah Wilts and Hilde Haider (sarah.wilts@uni-koeln.de, University of Cologne) submitted to the journal Psychological Research.

In this document the variable names and levels of the Excel file „Experiment2_training_data set“ are explained. The Excel file contains the raw data set of the training phase of the serial reaction time task.

subject_number	The numbers of the subjects from 1 to 64. The data of the participants 48 and 58 were not recorded due to technical issues.
condition	The participants were randomly assigned to the color-test condition (1) or the shape-test condition (2). The test-phase was the same for both conditions.
block	The training phase consisted of 7 blocks.
trial_total	The whole training phase consisted of 623 trials.
trial	Each block consisted of 89 trials.
color target	This column indicates the color of the color target. (1 = red, 2 = blue, 3 = green, 4 = orange, 5 = magenta, 6 = cyan).
shape target	This column indicates the shape of the shape target (1 = triangle, 2 = diamond, 3 = circle, 4 = arch, 5 = cross, 6 = star).
color deviant	In this column, deviant colors are indicated (1).
shape deviant	In this column, deviant shapes are indicated (1).
amot	This column indicates the correct response key (1 =spacebar, 2 = none).
answ	This column indicates the actual response key presses of the participants (1 = spacebar, 2 = none).
error	In this column, an erroneous trial is indicated (1).

response time	The computer measured the response times in milliseconds.
miss	This column indicates whether one of the deviant targets was missed by the participant (1).
false alarm	This column indicates whether a participant confounded a normal target for a deviant target (1).
color miss	This column indicates specifically whether a deviant color was missed by the participant (1).
shape miss	This column indicates specifically whether a deviant shape was missed by the participant (1).
